# Dexmedetomidine as an anesthetic adjunct is associated with reduced complications and cardiac intensive care unit length of stay after heart valve surgery

**DOI:** 10.1186/s12871-023-02227-5

**Published:** 2023-08-05

**Authors:** Zhi-Wei Fan, Yu-Xian Tang, Tuo Pan, Hai-Tao Zhang, He Zhang, Da-Liang Yan, Dong-Jin Wang, Kai Li

**Affiliations:** 1 Department of Cardio-Thoracic Surgery, Nanjing Drum Tower Hospital Clinical College of Nanjing University of Chinese Medicine, Number 321 Zhongshan Road, Nanjing, 210008 Jiangsu China; 2 Department of Cardio-Thoracic Surgery, Nanjing Drum Tower Hospital, Chinese Academy of Medical Science & Peking Union Medical College, Nanjing, 210008 Jiangsu China; 3 Department of Cardio-Thoracic Surgery, Nanjing Drum Tower Hospital Clinical College of Nanjing Medical University, Nanjing, 210008 Jiangsu China; 4 Department of Cardio-Thoracic Surgery, Nanjing Drum Tower Hospital, The Affiliated Hospital of Nanjing University Medical School, Nanjing, 210008 Jiangsu China

**Keywords:** Dexmedetomidine, Postoperative complications, Cardiac intensive care unit, Heart valve surgery, Anesthetic adjunct, Risk factors

## Abstract

**Background:**

We sought to explore the relationship between dexmedetomidine as an anesthetic adjuvant in cardiac surgery and postoperative complications and length of stay (LOS) in the cardiac intensive care unit (CICU).

**Methods:**

We conducted a retrospective study of patients aged 18 years and older who underwent heart valve surgery between October 2020 and June 2022. The primary endpoint of the study was major postoperative complications (cardiac arrest, atrial fibrillation, myocardial injury/infarction, heart failure) and the secondary endpoint was prolonged CICU LOS (defined as LOS > 90th percentile). Multivariate logistic regression analysis was performed for variables that were significant in the univariate analysis.

**Results:**

A total of 856 patients entered our study. The 283 patients who experienced the primary and secondary endpoints were included in the adverse outcomes group, and the remaining 573 were included in the prognostic control group. Multivariate logistic regression analysis revealed that age > 60 years (odds ratio [OR], 1.68; 95% confidence interval [CI], 1.23–2.31; *p* < 0.01), cardiopulmonary bypass (CPB) > 180 min (OR, 1.62; 95% CI, 1.03–2.55; *p* = 0.04) and postoperative mechanical ventilation time > 10 h (OR, 1.84; 95% CI, 1.35–2.52; *p* < 0.01) were independent risk factors for major postoperative complications; Age > 60 years (OR, 3.20; 95% CI, 1.65–6.20; *p* < 0.01), preoperative NYHA class 4 (OR, 4.03; 95% CI, 1.74–9.33; *p* < 0.01), diabetes mellitus (OR, 2.57; 95% CI, 1.22–5.41; *p* = 0.01), Intraoperative red blood cell (RBC) transfusion > 650 ml (OR, 2.04; 95% CI, 1.13–3.66; *p* = 0.02), Intraoperative bleeding > 1200 ml (OR, 2.69; 95% CI, 1.42–5.12; *p* < 0.01) were independent risk factors for prolonged CICU length of stay. Intraoperative use of dexmedetomidine as an anesthetic adjunct was a protective factor for major complications (odds ratio, 0.51; 95% confidence interval, 0.35–0.74; *p* < 0.01) and prolonged CICU stay. (odds ratio, 0.37; 95% confidence interval, 0.19–0.73; *p* < 0.01).

**Conclusions:**

In patients undergoing heart valve surgery, age, duration of cardiopulmonary bypass, and duration of mechanical ventilation are associated with major postoperative complication. Age, preoperative NYHA classification 4, diabetes mellitus, intraoperative bleeding, and RBC transfusion are associated with increased CICU length of stay. Intraoperative use of dexmedetomidine may improve such clinical outcomes.

## Background

Heart valve disease has become one of the most important factors affecting human health. Cardiac surgery is associated with a high risk of cardiovascular and other complications [1]. Perioperative myocardial injury/infarction, arrhythmias, and heart failure are the most common complications following heart valve surgery, and these complications can lead to decreased quality of life, increased intensive care and hospitalization time, and health care costs for patients. Reducing perioperative complications is key to improving the prognosis of patients undergoing heart valve surgery [2, 3].

Dexmedetomidine is a potent, highly selective alpha-2 adrenoceptor agonist with sedative, analgesic, anxiolytic, anti-sympathetic and opioid-sparing properties [4, 5]. A number of studies have shown that dexmedetomidine is a useful adjunct to cardiac anesthesia [6, 7]. Dexmedetomidine acts as an anesthetic adjuvant, reducing the need for opioids, inhalation anesthetics and intravenous anesthetics [8], and its anti-sympathetic activity reduces myocardial oxygen consumption by decreasing metabolism and preventing tachycardia, thereby reducing the incidence of postoperative complications in cardiac surgery, including myocardial ischemia [8, 9]. Therefore, in addition to investigating the more definitive endpoint of myocardial injury/infarction, this study examined the potential impact of dexmedetomidine on other primary endpoints, such as cardiac arrest during the postoperative period in patients undergoing cardiac surgery, congestive heart failure (CHF), and atrial fibrillation. Several studies have reported a reduction in CICU residence time with perioperative dexmedetomidine [10–12], suggesting that dexmedetomidine may promote rapid recovery of cardiac function in patients [12], but it is unknown whether the use of dexmedetomidine during maintenance of anesthesia for cardiac surgery has such an effect. Therefore, the specific aim of this study was to assess whether the intraoperative use of dexmedetomidine as an anesthetic adjuvant was associated with a reduced CICU length of stay and a lower rate of major postoperative complications in patients undergoing heart valve surgery. In addition, we will evaluate risk factors for poor prognosis in this group of patients.

## Method

### Study design and settings

Ethical approval for this retrospective observational study was obtained from the Medical Ethics Committee of Nanjing Drum Tower Hospital, Nanjing University School of Medicine (2020–249-01), and was conducted in accordance with the principles of the Declaration of Helsinki. Retrospective analysis of data collected between October 2020 and June 2022, which were mainly obtained from the hospital electronic medical record system, anesthesia system, and nursing system. Medical Ethics Committee of Nanjing Drum Tower Hospital, Nanjing University School of Medicine waived the need for written informed consent due to the retrospective nature of the study.

We collected perioperative patient data from the electronic medical record system, including demographic information, patient history, medical record information, preoperative risk factors, preoperative medications, intraoperative data, major postoperative complications, and CICU length of stay. The inclusion criteria were as follows: 1) age ≥ 18 years; 2) heart valve surgery performed under cardiopulmonary bypass; 3) ASA score III-V. The exclusion criteria were as follows: 1) those with severe preoperative hepatic, renal, pulmonary, or cerebral dysfunction; 2) emergency surgery, robotic procedures, and procedures requiring deep hypothermic arrest of circulation; 3) those with postoperative loss of follow-up and missing data.

### Definition

The follow-up period in this study was the period of CICU stay.The primary endpoint of the study was major postoperative complications (cardiac arrest, atrial fibrillation, myocardial injury/infarction, heart failure). Cardiac arrest (as opposed to death without resuscitation) was defined as the loss of circulation that prompted resuscitation by chest compressions, defibrillation, or a combination of both [13]. Postoperative serum cTnT above the 99th percentile upper reference limit (URL) was defined as myocardial injury, or myocardial infarction if the same was combined with one of the following conditions: 1) Symptoms of myocardial ischaemia; 2) New ischaemic ECG changes; 3) Development of pathological Q waves [14]; Heart failure was defined as a decrease in cardiac output and/or an increase in intracardiac pressure characterized by a range of symptoms (dyspnea, dyspnea, lower extremity swelling) and signs (elevated jugular venous pressure, pulmonary congestion), it is accompanied by elevated serum BNP [15]. Atrial fibrillation was defined as the loss of regular and orderly atrial electrical activity on postoperative bedside ECG monitors, which was replaced by rapid and disorganized flutter waves accompanied by an irregular and rapid heart rate. The secondary endpoint was prolonged CICU LOS, defined as LOS > 6 days (90th percentile). Patients who experienced the primary and secondary endpoints were entered into the adverse outcomes group, and the remaining patients were included in the prognosis control group.

### Surgical techniques and anesthetic procedures

All patients were treated with a median sternotomy and cardiopulmonary bypass (CPB). The ascending aorta was cannulated with a cannula appropriate to the patient's size. A separate cannula was used for intravenous cannulation in the superior and inferior vena cava [16].

All patients were induced with midazolam 0.1 mg-kg-1, etomidate 0.2 mg-kg-1, sufentanil 10 μg-kg-1, and vecuronium 0.2 mg-kg-1 intravenously. Anesthesia was maintained entirely by intravenous anesthesia, and the use of dexmedetomidine was determined by the anesthesiologist based on his/her experience, and the pumping rate of dexmedetomidine varies from 0.2 to 0.7 μg/kg/h. Depending on the desired depth of anesthesia, intraoperative anesthesia was maintained with propofol 4–6 mg-kg-1- h-1 and vecuronium 1–2 μg-kg-1- min-1. During perioperative ventilation, the ventilator parameters were set at a tidal volume of 6–8 ml-kg-1 and a frequency of 10–12 breaths-min-1 to maintain end-expiratory CO2 (ETCO2) at 35–45 mm Hg.

### Sample size and statistical analysis

According to the retrospective analysis, the incidence of postoperative cardiac complications in our center is between 30 and 40%, and in order to calculate the sample size, we need to make the following settings in the pmsampsize R package. Using a primary endpoint incidence rate of 40% as the basis, and assuming an α (type I error probability) of 0.05 and a δ (allowable error) of 10% [17, 18], the estimated sample size was 342 cases.In order to further improve the reliability of the study, we ultimately planned to include 856 patients for the following analysis.

IBM SPSS statistical software was used (Statistics for Windows, version 26, IBM Corporation, Armonk, NY, USA). The normality of the distribution of continuous variables was assessed by the Shapiro–Wilk test. Normally distributed variables are expressed as the mean ± standard deviation and were compared using Student’s t-test. Nonparametric continuous variables are expressed as medians (interquartile ranges (IQRs)) and were compared using the Mann–Whitney U test. Categorical data were compared using the chi-square test or Fisher’s exact test.

For preoperative demographic information such as age、gender、body mass index 、 NYHA class、 past medical history and history of medication use; Intraoperative surgical information such as surgical procedure、ASA class、 time to cardiopulmonary bypass、 blood transfusion and bleeding、 hemodynamic indices and use of vasoactive medications; Postoperative 24-h blood gas analysis and duration of mechanical ventilation, these variables were included in the univariate analyses. We combined the optimal thresholds determined by ROC curves, previous studies reported in the literature, and clinicians' diagnostic experience to stratify some continuous variables for better study.All variables that were statistically significant (*P* < 0.05) in univariate analyses, as well as those considered potentially significant by clinicians, were included in multivariate logistic regression models. In this study, intraoperative dexmedetomidine was added to the model as an independent factor to assess the relationship between dexmedetomidine use and clinical outcomes.

## Results

### Patient characteristics

**Fig. 1:**
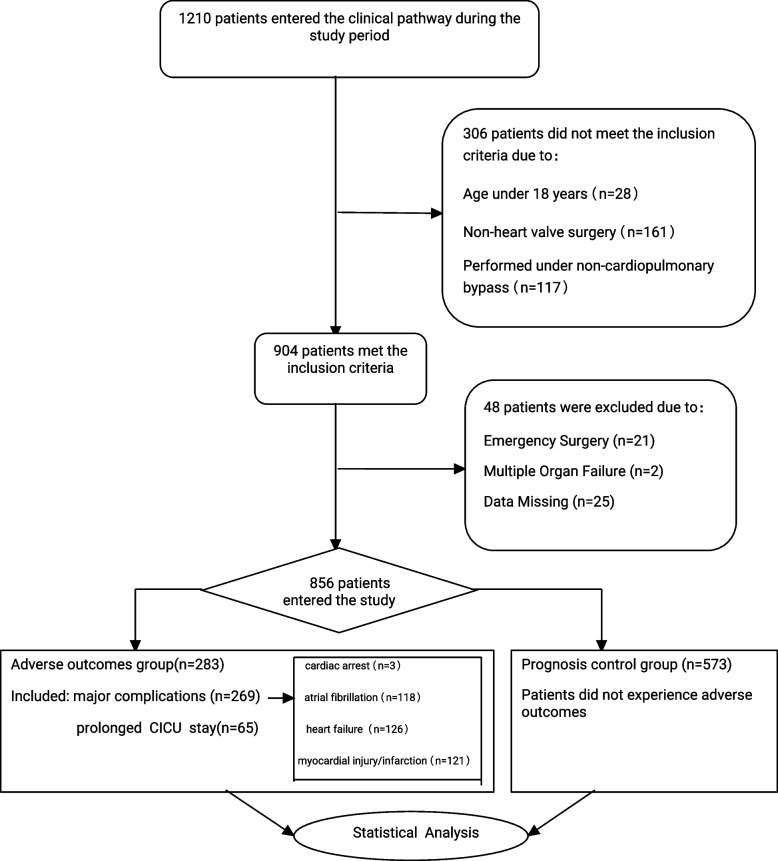
Consort diagram of patient screening and allocation

During the study period, 1,210 patients were admitted to our center, and 856 patients were eventually entered into our study based on inclusion and exclusion criteria (Fig. 1). The 283 patients who experienced the primary and secondary endpoints were included in the adverse outcomes group, of which 269 patients (31.43%) experienced the primary endpoint, including cardiac arrest (*n* = 3) 、atrial fibrillation (*n* = 118) 、myocardial injury/infarction (*n* = 121) 、heart failure (*n* = 126). 65 patients (7.59%) experienced the secondary endpoint, and the remaining 573 were included in the prognostic control group, which is consistent with previous reports [1, 19].

There were no significant differences between the two groups in terms of gender, weight, New York Heart Association (NYHA) classification, preoperative left ventricular ejection fraction, previous medical history, previous cardiac surgery history, or preoperative medication use (*P* > 0.05). Compared with the control group, Patients in the adverse outcomes group were older(median: 66 years, IQR: 58–70 years vs. median: 61 years, IQR: 53–69 years, *P* < 0.001) (Table 1).

**Table 1:** Demographic and clinical characteristics

Variable	Adverse outcomes(*n* = 283)	Control(*n* = 573)	*p* value
Age(year)	66 (58–70)	61 (53–69)	< 0.001
Gender(male,%)	174 (61.5)	329 (57.4)	0.255
BMI(kg/m^2^)	23.4 (21.5–25.6)	23.5 (21.6–25.5)	0.813
Preoperative LVEF (%)	54 (46–57)	55(48–57)	0.191
NYHA(n,%)			0.261
II	109 (38.5)	222 (38.7)	
III	153 (54.1)	324 (56.5)	
IV	21 (7.4)	27 (4.7)	
Previous history of cardiac surgery(n,%)	30 (10.6)	44 (7.7)	0.152
Previous medical history(n,%)			
Heart failure	3 (1.1)	5 (0.9)	0.789
Atrial fibrillation	29 (10.2)	65 (11.3)	0.629
Hypertension	116 (41.0)	231 (40.3)	0.850
Diabetes mellitus	33 (11.7)	53 (9.2)	0.270
COPD	16 (5.7)	36 (6.3)	0.717
Chronic liver disease	6 (2.1)	10 (1.7)	0.703
Chronic renal disease	9 (3.2)	10 (1.7)	0.180
Coronary disease	38 (13.4)	64 (11.2)	0.337
Smoking	29 (10.2)	43 (7.5)	0.174
Excessive alcohol	29 (10.2)	42 (7.3)	0.145
Medications(n,%)			
Statins	11 (3.9)	12 (2.1)	0.127
Hypoglycemic drug	31 (11.0)	51 (8.9)	0.337
Antiplatelet	11 (3.9)	15 (2.6)	0.309
Anticoagulant	9 (3.2)	23 (4.0)	0.545
Calcium channel blocker	66 (23.3)	111 (19.4)	0.179
ACE inhibitor or ARB	51 (18.0)	112 (19.5)	0.593
Diuretic	16 (5.7)	34 (5.9)	0.869
b-blocker	29 (10.2)	56 (9.8)	0.827

*BMI* Body Mass Index, *NYHA* New York Heart Association, *COPD* Chronic obstructive pulmonary disease, *ACEI* Angiotensin-converting-enzyme inhibitor, *ARB* Angiotensin receptor blocker

### Intraoperative and postoperative variables

Intraoperative and postoperative variables between adverse outcomes group and control group shown in Table 2. There was no significant difference between the two groups in the proportion of patients with the type of cardiac surgery, ASA classification (*P* > 0.05). In contrast, Patients in the adverse outcomes group had significantly longer operative time(median: 295 min, IQR: 250–360 min vs. median: 270 min, IQR: 230–325 min, *P* < 0.001), cpb time(median: 150 min, IQR: 119–188 min vs. median: 134 min, IQR: 107–163 min, *P* < 0.001), and aortic block time(median: 110 min, IQR: 85–144 min vs. median: 98 min, IQR: 79–126 min, *P* < 0.001). Patients in the adverse outcomes group had more intraoperative bleeding(median: 800 ml, IQR: 600–1200 ml vs. median: 800 ml, IQR: 600–1100 ml, *P* = 0.005), and there was no significant difference in the amount of red blood cell infusion as well as urine volume(*P* > 0.05). Hemodynamically, patients in the adverse outcomes group had a significantly higher heart rate than the control group at the end of surgery(median: 93 bpm, IQR: 85–101 bpm vs. median: 90 bpm, IQR: 80–98 bpm, *P* < 0.001). Regarding intraoperative drug use, patients in the CONTROL group used a higher proportion of dexmedetomidine(84.1 vs. 74.9, *P* = 0.001) and significantly reduced the use of milrinone(median: 2.6 mg, IQR: 2.1–3.2 mg vs. median: 2.7 mg, IQR: 2.1–3.8 mg, *P* = 0.023) and dopamine(median: 42.2 mg, IQR: 42.0–53.6 mg vs. median: 45.0 mg, IQR: 35.1–62.7 mg, *P* = 0.007).

**Table 2:** Intraoperative and postoperative variables

	Adverse outcomes(n = 283)	Control(n = 573)	*P* value
Intraoperative variables			
Type of cardiac surgery (n, %)			0.442
AVR	94 (33.2)	205 (35.8)	
MVR	83 (29.3)	164 (28.6)	
DVR	45 (15.9)	66 (11.5)	
CABG + valvular surgery	13 (4.6)	28 (4.9)	
Valvular + other aortic surgery	48 (17.0)	110 (19.2)	
ASA Classification > 3 (n,%)	249 (88.0)	477 (83.2)	0.069
Operation time( minutes)	295 (250–360)	270 (230–325)	< 0.001
CPB( minutes)	150 (119–188)	134 (107–163)	< 0.001
ACC(minutes)	110 (85–144)	98 (79–126)	< 0.001
Intraoperative bleeding(ml)	800 (600–1200)	800 (600–1100)	0.005
Intraoperative RBC			
transfusion(ml)	450 (0–1050)	350 (0–904)	0.135
Urine volume(ml)	800 (500–1300)	800 (500–1400)	0.544
Maximum HR(bpm)			
Preoperative	65 (55–80)	68 (59–80)	0.544
End of surgery	93 (85–101)	90 (80–98)	< 0.001
Maximum MAP(mmHg)			
Preoperative	80 (73–86)	80 (73–87)	0.175
End of surgery	73 (67–79)	74 (68–80)	0.058
Intraoperative drugs			
Dextrmetomidine(n,%)	212(74.9)	482(84.1)	0.001
Milrinone(mg)	2.7 (2.1–3.8)	2.6 (2.1–3.2)	0.023
Norepinephrine(mg)	1.4 (1.1–1.9)	1.3 (1.1–1.7)	0.206
Dopamine(mg)	45.0 (35.1–62.7)	42.2 (34.0–53.6)	0.007
Postoperative variables			
Blood gas analysis within 24 h			
Sp02(%)	99 (98–100)	99 (98–100)	0.956
PH	7.40 (7.38–7.43)	7.40 (7.38–7.42)	0.048
PaCO2(mmHg)	38.6 (35.0–41.7)	39.6 (37.3–42.0)	0.002
PaO2(mmHg)	108.1(89.6–142.9)	107 (90.6–126.85)	0.641
Hb(mmol/L)	10.2 (9.2–11.0)	10.4 (9.6–11.3)	0.001
K + (mmol/L)	4.4 (4.2–4.7)	4.5 (4.2–4.7)	0.377
Lac(mmol/L)	1.7 (1.3–2.5)	1.6 (1.2–2.3)	0.212
Mechanical ventilation time (hours)	13 (7–20)	7 (5–14)	< 0.001
CICU time (days)	4 (3–6)	3 (3–4)	< 0.001
Cardiac arrest(n, %)	3 (1.1)	0 (0)	0.014
Atrial fibrillation (n, %)	118 (41.7)	0 (0)	< 0.001
Myocardial injury/infarction(n, %)	121 (42.8)	0 (0)	< 0.001
Heart failure(n, %)	126 (44.5)	0 (0)	< 0.001

*AVR* Aortic valve replacement/repair, *MVR* Mitral valve replacement/repair, *DVR* Double valve replacement/repair, *CABG* Coronary artery bypass grafting, *ASA* American society of Aneshesiologists, *CPB* Cardiopulmonary Bypass, *ACC* Aortic Cross Clamp, *RBC* Red Blood Cell, *HR* Heart Rate, *MAP* Mean Artery Pressure, *SpO2* Peripheral capillary oxygen saturation, *PH* Potential of Hydrogen, *PaCO2* Partial pressure of Carbon dioxide, *PaO2* Partial pressure of Oxygen, *Hb* Hemoglobin, *Lac* Lactobacillus acidophilus, *CICU* Cardiac intensive care unit

Patients in the Adverse outcomes group had a lower Partial pressure of carbon dioxide(median: 38.6 mmHg, IQR: 35.0–41.7 mmHg vs. median: 39.6 mmHg, IQR: 37.3–42.0 mmHg, *P* = 0.002) and Hemoglobin(median: 10.2 mmol/L, IQR: 9.2–11.0 mmol/L vs. median: 10.4 mmol/L, IQR: 9.6–11.3 mmol/L, *P* = 0.001) on blood gas analysis in the first 24 h after surgery. In addition, patients in the adversarial outcomes group had a longer duration of postoperative mechanical ventilation(median: 13 h, IQR: 7–20 h vs. median: 7 h, IQR: 5–14 h, *P* < 0.001).

We combined the optimal thresholds determined by ROC curves (Table 3), previous studies reported in the literature, and clinicians' diagnostic experience to stratify some continuous variables and conduct univariate analysis of the two sub-groups.

**Table 3:** Area under the ROC curve for continuous variables

Variable	AUC	95%CI	Cut-off value	Sensitivity	Specificity	*p* value
Age	0.591	0.552–0.630	59.5	0.693	0.478	< 0.001
Intraoperative bleeding	0.558	0.518–0.599	1205.0	0.237	0.852	0.005
Intraoperative RBC transfusion	0.530	0.489–0.572	637.5	0.424	0.668	0.147
CPB	0.598	0.557–0.639	156.5	0.470	0.707	< 0.001
Intraoperative Milrinone	0.548	0.506–0.590	3.8	0.251	0.873	0.022
Intraoperative dopamine	0.556	0.514–0.599	62.6	0.251	0.878	0.007
Max HR at the end of surgery	0.585	0.544–0.625	97.5	0.420	0.710	< 0.001
Postoperative Mechanical ventilation	0.649	0.609–0.689	7.0	0.714	0.522	< 0.001

*AUC* Area Under Curve, *CI* Confidence Interval, *CPB* Cardiopulmonary Bypass, *HR* Heart Rate, *RBC* Red Blood Cell

**Fig. 2:**
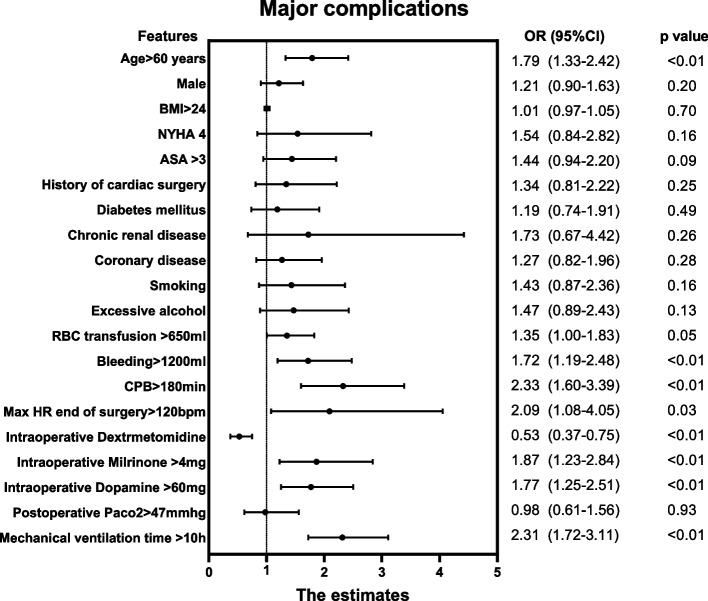
Univariate analysis of major complications. BMI: Body Mass Index, NYHA: New York Heart Association, ASA: American society of Aneshesiologists, CPB: Cardiopulmonary Bypass, RBC:Red Blood Cell, HR:Heart Rate, PaCO2: Partial pressure of Carbon dioxide, OR:Odds ratio, CI:Confidence Interval

In the Univariate analysis in patients with major complications, age > 60 years (OR:1.79, 95%CI:1.33–2.42, *P* < 0.01)、bleeding > 1200 ml (OR:1.72, 95%CI: 1.19–2.48, *P* < 0.01)、CPB > 180 min(OR: 2.33, 95%CI: 1.60–3.39, *P* < 0.01)、intraoperative dextrmetomidine(OR: 0.53, 95%CI: 0.37–0.75, *P* < 0.01) 、intraoperative Milrinone > 4 mg(OR: 1.87, 95%CI: 1.23–2.84, *P* < 0.01)、intraoperative dopamine > 60 mg(OR: 1.77, 95%CI: 1.25–2.51, *P* < 0.01)、postoperative mechanical ventilation time > 10 h (OR: 2.31, 95%CI: 1.72–3.11, *P* < 0.01) increased the rate of adverse events. The detailed variables for the univariate analysis are shown in Fig. 2, with a forest plot applied for easy reading.

**Fig. 3:**
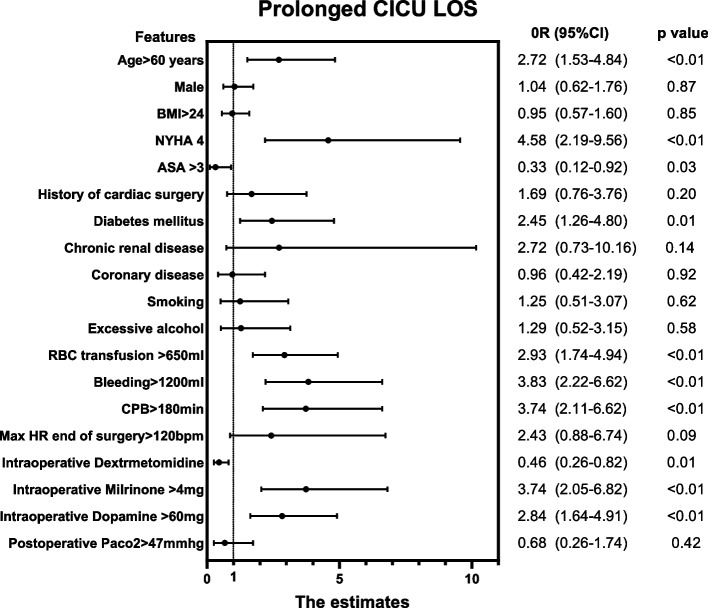
Univariate analysis of prolonged CICU LOS. BMI: Body Mass Index, NYHA: New York Heart Association, ASA: American society of Aneshesiologists, CPB: Cardiopulmonary Bypass, RBC:Red Blood Cell, HR:Heart Rate, PaCO2: Partial pressure of Carbon dioxide,CICU LOS: Cardiac Intensive Care Unit Length Of Stay,OR:Odds ratio, CI:Confidence Interval

In the Univariate analysis in patients with prolonged CICU LOS, age > 60 years (OR:2.72, 95%CI:1.53–4.84, *P* < 0.01)、NYHA classification 4 (OR:4.58, 95%CI: 2.19–9.56, *P* < 0.01)、diabetes mellitus(OR:2.45, 95%CI: 1.26–4.80, *P* = 0.01)、intraoperative RBC transfusion > 650 ml (OR: 2.93, 95%CI: 1.74–4.94, *P* < 0.01) 、bleeding > 1200 ml (OR:3.83, 95%CI: 2.22–6.62, *P* < 0.01)、CPB > 180 min(OR: 3.74, 95%CI: 2.11–6.62, *P* < 0.01)、intraoperative dextrmetomidine(OR: 0.46, 95%CI: 0.26–0.82, *P* = 0.01) 、intraoperative Milrinone > 4 mg(OR: 3.74, 95%CI: 2.05–6.82, *P* < 0.01)、intraoperative dopamine > 60 mg(OR: 2.84, 95%CI: 1.64–4.91, *P* < 0.01) increased the prolonged CICU LOS. The detailed variables for the univariate analysis are shown in Fig. 3.

### The results of multi-factor regression analysis

Multivariate logistic regression analysis revealed that age > 60 years (odds ratio [OR], 1.68; 95% confidence interval [CI], 1.23–2.31; *p* < 0.01), cardiopulmonary bypass( CPB) > 180 min (OR, 1.62; 95% CI, 1.03–2.55; *p* = 0.04) and postoperative mechanical ventilation time > 10 h (OR, 1.84; 95% CI, 1.35–2.52; *p* < 0.01) were independent risk factors for major postoperative complications; Age > 60 years (OR, 3.20; 95% CI, 1.65–6.20; *p* < 0.01), preoperative NYHA class 4 (OR, 4.03; 95% CI, 1.74–9.33; *p* < 0.01), diabetes mellitus (OR, 2.57; 95% CI, 1.22–5.41; *p* = 0.01),Intraoperative RBC transfusion > 650 ml (OR, 2.04; 95% CI, 1.13–3.66; *p* = 0.02), Intraoperative bleeding > 1200 ml (OR, 2.69; 95% CI, 1.42–5.12; *p* < 0.01)were independent risk factors for prolonged CICU length of stay. Intraoperative use of dexmedetomidine as an anesthetic adjunct was a protective factor for major complications (odds ratio, 0.51; 95% confidence interval, 0.35–0.74; *p* < 0.01)and prolonged CICU stay. (odds ratio, 0.37; 95% confidence interval, 0.19–0.73; *p* < 0.01) (Table 4).

**Table 4:** Multivariate analysis of patients with major complications and prolonged CICU LOS

Variable	Major complications		Prolonged CICU LOS	
	Odds ratio ( 95%CI)	*p* value	Odds ratio ( 95%CI)	*p* value
Age > 60 years	1.68(1.23–2.31)	< 0.01	3.20(1.65–6.20)	< 0.01
NYHA classification 4			4.03(1.74–9.33)	< 0.01
Diabetes mellitus			2.57(1.22–5.41)	0.01
ASA classification > 3	1.33(0.85–2.08)	0.21	2.49(0.82–7.54)	0.11
Intraoperative RBC transfusion > 650 ml	1.16(0.84–1.60)	0.38	2.04(1.13–3.66)	0.02
Intraoperative bleeding > 1200 ml	1.19(0.79–1.80)	0.40	2.69(1.42–5.12)	< 0.01
Max HR at the end of surgery > 120 bpm	1.68(0.84–3.39)	0.15	1.94(0.62–6.12)	0.26
CPB > 180 min	1.62(1.03–2.55)	0.04	1.91(0.86–4.26)	0.11
Intraoperative Dextrmetomidine	0.51(0.35–0.74)	< 0.01	0.37(0.19–0.73)	< 0.01
Intraoperative Milrinone > 4 mg	1.04(0.53–2.03)	0.91	1.92(0.57–6.45)	0.29
Intraoperative dopamine > 60 mg	1.27(0.72–2.24)	0.42	1.21(0.40–3.62)	0.73
Postoperative Mechanical ventilation time > 10 h	1.84(1.35–2.52)	< 0.01		

*CICU LOS* cardia$c intensive care unit length of stay, *ASA* American society of Aneshesiologists, *CPB* Cardiopulmonary Bypass, *HR* Heart Rate, *RBC* Red Blood Cell, *PaCO2* Partial pressure of Carbon dioxide, *CI* Confidence Interval

## Discussion

The Society of Thoracic Surgeons (STS) reports that the incidence of major complications of valve and coronary artery bypass grafting (CABG) is as high as 30.1%. These complications can lead to increased mortality and longer hospital stays [1]. In our center's analysis of patients undergoing heart valve surgery, the incidence of major complications after heart valve surgery was 31%, which is roughly the same as the data reported for STS. We found that intraoperative dexmedetomidine use was associated with a lower incidence of major postoperative complications and reduced CICU LOS, and that older age, prolonged duration of cardiopulmonary bypass and postoperative mechanical ventilation were risk factors for major postoperative complication. Older age, preoperative NYHA class 4, diabetes mellitus, intraoperative bleeding, and RBC transfusion were risk factors for increased CICU length of stay.As many studies have shown, age is an important risk factor for morbidity and mortality in patients undergoing cardiac surgery [20–22]. Performing cardiac surgery in the elderly is challenging due to decreased functional reserve capacity and more coexisting disease than in younger patients [23]. The rate of perioperative complications and mortality is relatively high in patients over 80 years of age, but survival is stable in the medium term [3]. However, Lin's a systematic review showed that cardiac surgery outcomes were related to patient frailty and not to actual age [24]. In our study, patients in the adverse outcomes group were older, and multivariate regression analysis showed that age over 60 years was a risk factor for morbidity in patients undergoing heart valve surgery, and a corresponding increase in CICU stay.Therefore, we recommend that a more gentle procedure should be performed on elderly patients after a more careful selection of surgical options.

Cardiopulmonary bypass (CPB) provides blood and oxygen to the body's organs and tissues when the heart stops working, allowing direct intracardiac surgery to be performed successfully and maintaining the body's metabolism [25, 26]. However, the ensuing myocardial injury and inflammation associated with ischemia–reperfusion directly affect the recovery of postoperative cardiac function [12]. Chen et al. also showed in a previous study that cardiac surgery using CPB procedures was associated with ischemia/reperfusion injury, and that ischemia/reperfusion injury was strongly associated with reversible post-ischemic cardiac dysfunction and irreversible cardiomyocyte death [27]. In our study, prolonged cardiopulmonary bypass time was associated with an increased incidence of complications after cardiac surgery; therefore, we need to provide myocardial protection during cardiac surgery CPB by a series of measures to reduce oxygen consumption by cardiomyocytes to adapt to transient ischemia.

Atrial arrhythmias are the most common complication after cardiac surgery [28]. The incidence of tachycardia and arrhythmias is common during surgery due to procedures involving tracheal intubation, which increases sympathetic and sympatho-renal activity. Increased sympatho-renal activity leads not only to tachycardia and arrhythmias, but also to increased myocardial oxygen consumption and local ischemia [29]. We believe that for patients undergoing heart valve surgery, early postoperative extubation to reduce the duration of mechanical ventilation may be beneficial, but further studies and validation are needed to support this idea.

Several studies have shown that comorbid diabetes mellitus before cardiac surgery was associated with increased intensive care unit and hospital LOS in both children and adults [30–32], and we confirmed this risk factor in our regression analysis of secondary endpoints. This may be due to the fact that diabetic patients have more infections, heart failure and more difficult surgical wound healing [33]. This also shows the importance of carrying out perioperative diabetes management.

Bleeding and red blood cell transfusion are common in cardiac surgery, and any definition of bleeding as well as transfusion is limited and arbitrary [34], We stratified intraoperative bleeding and red blood cell transfusion in our study and found that bleeding > 1200 ml, red blood cell transfusion > 650 ml was associated with prolonged CICU LOS. Andrew E Newcomb et al. in a prospective cohort study found that excessive bleeding from cardiac surgery and excessive red blood cell transfusions resulted in longer hospital stays and higher treatment costs [35]. Nawwar Al-Attar et al. in a retrospective study also demonstrated that patients with bleeding complications in cardiac surgery spent more days in intensive care [36]. Some studies have suggested that blood transfusion worsens the systemic inflammatory response syndrome triggered by CPB with secondary inflammatory response, thus increasing the poor prognosis [37].

Multivariate regression analysis showed that intraoperative use of dexmedetomidine was a protective factor. Many studies have shown that dexmedetomidine reduces myocardial complications after cardiovascular surgery in adults, with a reduction in postoperative myocardial injury and arrhythmic events in patients using dexmedetomidine [28, 38]. Because it reduces the release of cytokines, inhibits the inflammatory response and reduces ischemia–reperfusion injury, thus contributing to organ protection [39, 40]. Han, Y et al. found that intraoperative application of a loading dose of dexamethasone combined with propofol during maintenance of anesthesia reduced heart rate and thus oxygen consumption by cardiac myocytes [41]. In addition, several studies have reported that perioperative use of dexmedetomidine reduced the duration of intensive care and tracheal intubation and the incidence of short-term mortality after cardiac surgery in adults [11, 12, 42, 43]. The elimination half-life of dexmedetomidine has been reported to be 2.1–3.1 h in healthy volunteers [44, 45], and age, body size, and hepatic impairment may have a significant effect on the pharmacokinetics of dexmedetomidine [46]. There are few pharmacokinetic studies of dexmedetomidine in cardiac surgery, and despite the short half-life of dexmedetomidine, its complete elimination in vivo is influenced by many factors, with hypoalbuminemia, end-organ damage, hemodynamic changes, and reduced cardiac output all potentially contributing to high inter-individual variability, especially in the ICU population [47, 48]. During the study period, the average postoperative CICU stay of patients undergoing cardiac surgery at our center was about 4 to 5 days, and to make the study more clinically instructive, we referred to previous studies that defined prolonged CICU LOS as longer than 6 days and used it as a secondary endpoint [49, 50], and the results surprised us, We found that dexmedetomidine as an anesthetic adjuvant was not only associated with a lower incidence of major postoperative complications, but also with a shorter CICU LOS. Therefore, we believe that dexmedetomidine may benefit patients undergoing heart valve surgery.

## Conclusion

In conclusion, in patients undergoing heart valve surgery, age, duration of cardiopulmonary bypass, and duration of mechanical ventilation are associated with major postoperative complication. Age, preoperative NYHA classification 4, diabetes mellitus, intraoperative bleeding, and red blood cell (RBC) transfusion are associated with increased CICU length of stay. Intraoperative use of dexmedetomidine may improve such clinical outcomes.

## Limitation

Our study has several limitations. First, this is a single-center, retrospective, non-randomized study, and many confounding factors may affect the reliability of our conclusions. Second, in our study, the number of patients with prolonged CICU LOS was much smaller than those with primary outcomes, and the short half-life of dexmedetomidine somewhat affects the reliability of our conclusions.The absence of other possible factors, such as the preoperative patient's EuroSCORE score, the type of intraoperative anesthetic drug, the dose used, and the timing of its administration, may have limited our findings. Again, the primary outcome is composed of four postoperative complications, an approach that improves statistical efficiency but may challenge the interpretation of results [51]. In regression analysis, continuous variables are converted to binary categorical variables, which may lead to loss of some information. Finally, because this study added the use of intraoperative dexmedetomidine as an independent factor to a multivariate logistic regression model that could not directly confirm the effect of this drug on certain clinical outcomes, further prospective, multicenter, randomized studies are needed in the future to confirm the benefits demonstrated in this study.

## Data Availability

The datasets used and/or analyzed during the current study are available from the corresponding authors on reasonable request.
